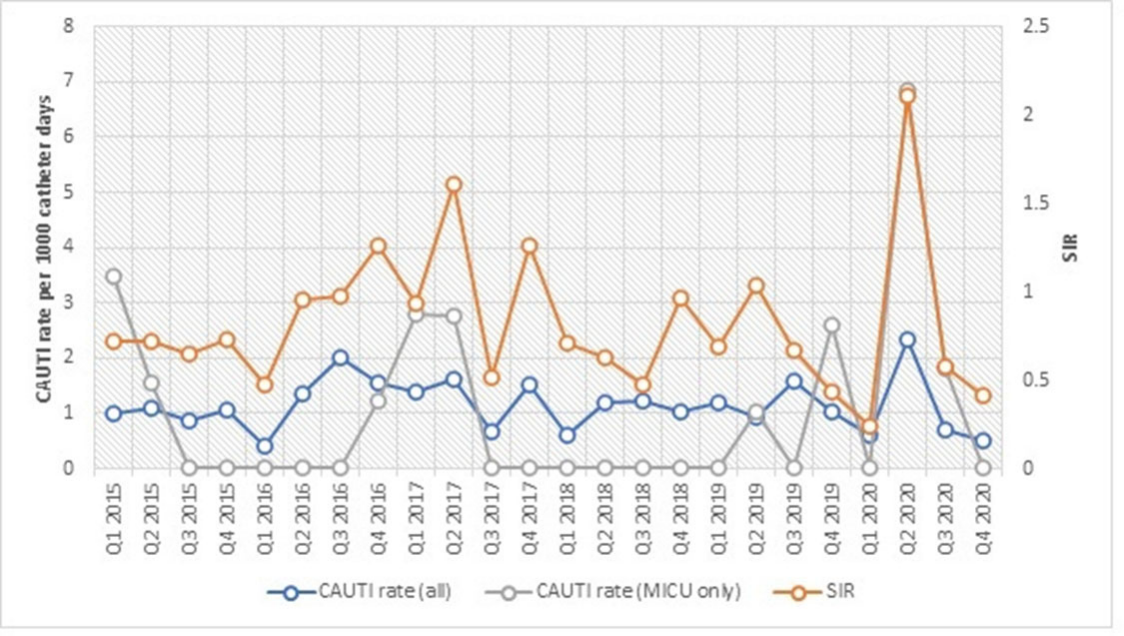# The Impact of Coronavirus Disease 2019 (COVID-19) Pandemic on Device-Associated Healthcare-Associated Infection

**DOI:** 10.1017/ash.2021.104

**Published:** 2021-07-29

**Authors:** Minji Kang, Sharen Henry, Elizabeth Thomas, Doramarie Arocha, Julie Trivedi

## Abstract

**Background:** The impact of the coronavirus disease 2019 (COVID-19) pandemic on healthcare-associated infection (HAI) is not yet known. Diversion of resources from traditional HAI surveillance and prevention efforts toward institutional COVID-19 response, along with decrease in patient contact due to fear or required quarantine or isolation, may have increased HAI rates. In contrast, increased compliance with hand hygiene and personal protective equipment may have decreased HAI rates. **Methods:** We sought to determine the impact of COVID-19 pandemic on healthcare-associated central-line–associated bloodstream infection (CLABSI) and catheter-associated urinary tract infection (CAUTI). CLABSI and CAUTI rates and standardized infection ratios (SIRs) reported to the NHSN from the first quarter of 2015 to the fourth quarter of 2020 were obtained for the entire facility and for the medical intensive care unit (MICU), which was converted during the pandemic to an intensive care unit solely for critically ill patients with COVID-19. Changes in CLABSI and CAUTI rates and SIRs before the pandemic (Q1 2015 to Q4 2019) and during the pandemic (Q1 2020 to Q4 2020) were assessed using an independent-sample *t* test. **Results:** The CLABSI rate was unchanged, with a mean (SD) of 0.64 (±0.34) CLABSIs per 1,000 central-line days before the pandemic and 0.72 (±0.22) during the pandemic (*P* = .62) (Figure 1). The SIR remained stable at 0.54 (±0.29) before and 0.96 (±0.59) during the COVID-19 pandemic (*P* = .25). However, CLABSI rate in MICU increased significantly from 0.92 (±1.00) to 2.75 (±1.00) (p < 0.01), along with SIR from 0.81 ± 0.89 to 2.53 ± 1.07 (p < 0.01) (Figure 1). CAUTI rate was unchanged with 1.17 ± 0.38 CAUTI per 1000 catheter days per quarter before, and 1.04 ± 0.87 during COVID-19 pandemic (p = 0.64). CAUTI SIR remained stable at 0.82 ± 0.31 before and 0.83 ± 0.86 during COVID-19 pandemic (p = 0.96). CAUTI rate in MICU was 0.78 ± 1.20 before and 2.17 ± 3.24 after COVID-19 pandemic (p = 0.45) (Figure 2). **Conclusions:** Although our institutional CLABSI and CAUTI rates and SIRs remained unchanged, our medical intensive care unit, which housed our critically ill patients with COVID-19, experienced significant increases in CLABSI rate and SIR. This finding is likely multifactorial in the setting of overextended nursing staff, use of prone position, and challenges of infection prevention efforts under isolation precautions.

**Funding:** No

**Disclosures:** None

**Figure 1.**
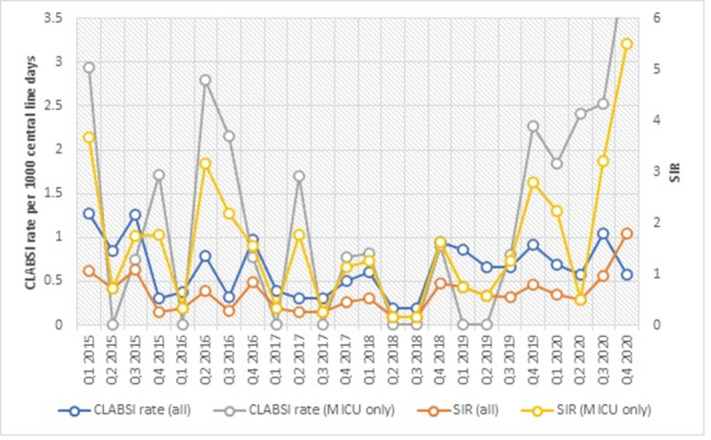

**Figure 2.**